# Rapid and Sensitive Analysis of Volatile Components of Different Parts of *Clausena lansium* by Ionic Liquid Based Headspace Gas Chromatography-Mass Spectrometry

**DOI:** 10.3390/molecules24010091

**Published:** 2018-12-27

**Authors:** Xiaowen He, Yinzheng Ma, Guohui Yi, Yingying Wen, Yunxia Zhang, Haiying Zhang, Lantong Zhang

**Affiliations:** 1 School of Pharmacy, Hebei Medical University, Shijiazhuang 050017, China; xiaowen_he@126.com; 2 Public Research Laboratory, Hainan Medical University, Haikou 571199, China; guohuiyi6@163.com (G.Y.); hnmuzyx@126.com (Y.Z.); hyzhang_xjtu2013@aliyun.com (H.Z.); 3 School of Public Health, Hainan Medical University, Haikou 571199, China; hbykdxmyz@163.com; 4 Key Laboratory of Translation Medicine Tropical Disease, Department of Ministry of Education, Hainan Medical University, Haikou 571199, China; wyy_418@163.com

**Keywords:** ionic liquid, headspace, volatile components, *Clausena lansium*, orthogonal experimental design, cluster analysis

## Abstract

A rapid and sensitive ionic liquid (IL) based headspace gas chromatography-mass spectrometry (HS-GC-MS) method was developed for analyzing volatile components in leaf, pericarp, and seed of *Clausena lansium* from different areas in Hainan Province, China. HS efficiencies were carefully investigated by using three ILs and water as matrix media. Extraction parameters, including equilibrium temperature, equilibrium time, and stirring rate had been evaluated and optimized by using an orthogonal design with OA_9_(3^3^) table. Under the optimized condition of IL-based HS-GC-MS, only 100 mg of sample and 2 mL of [Bmim][BF_4_] were needed to comprehensively and accurately analyze the volatile components in *Clausena lansium*. By utilizing a cluster analysis, six clusters were obtained for ninety components. This method was simpler, more rapid, and more sensitive when compared with previously reported methods for analyzing and identifying volatile components in *Clausena lansium.* The results may provide a theoretical basis for further exploitation of *Clausena lansium*.

## 1. Introduction

*Clausena lansium* (Lour.) Skeels, commonly known as Wampee, is a tropical species of the Rutaceae family. It has a long history of cultivation in southern China, especially in Hainan, Guangxi, Guangdong, Taiwan, etc. [1]. *Clausena lansium* has attracted great attention due to its extensive pharmacological benefits, including antioxidant, anticancer, antidiabetic, hepatoprotective, antinociceptive, nootropic, cerebroprotective, and anti-trichomonal effects [2,3,4,5]. In traditional Chinese medicine, the leaf, pericarp, and seed of *Clausena lansium* can be used for different diseases. The leaf of *Clausena lansium* is used for treating fever, cough, asthma, malaria, dermatological, and rheumatism, etc. When the fruit fully ripens around July, it is used for digestive disorders, gastro-intestinal diseases, and bronchitis [6], because it releases gastrointestinal gas, eliminates stagnation, dissipates heat, and relieves pain.

There are lots of volatile components in *Clausena lansium*, which play an important role in the above-mentioned benefits. Hydrodistillation is one of traditional extraction procedures for the pretreatment of volatile components. Previously, essential oils of *Clausena lansium* from China, Thailand, and Cuba have been studied by hydrodistillation followed by gas chromatography-mass spectrometry (GC-MS) [7,8,9]. However, extraction has some shortcomings, including the use of large quantity of samples, tedious process, and time-consuming operation. In addition, extraction is usually performed with an open system, which may lead to the loss of volatile components and cause the inaccuracy of the results. Recently, supercritical fluid extraction (SFE) has been applied to extract volatile components in *Clausena lansium* due to its high efficiency without chemical pollutions [10]. In addition, the extracted components can be well protected from oxygenation. The chemical constituents of volatile oil from fruits of *Clausena lansium* in Guangdong, China were studied. However, only a few components were detected because this pretreatment was not sensitive enough and time-consuming.

The headspace (HS) technique, with its simple installation, low cost, less pollution on the chromatographic system, fast, and sensitivity, has been increasingly applied for the pretreatment of volatile components. HS-GC-MS has been gaining increasing attention for the determination of volatile components in food, medicine, and environment samples [11,12]. Chokeprasert et al. [13] analyzed the volatile components of fresh *Clausena lansium* leaf, flesh, skin, and seed in Thailand by HS-GC-MS. This method was simple and sensitive, with negligible pollution on the detecting system. 1 g of each fresh sample was directly placed into a vial for analysis, no extra matrix media were used in the study and the matrix media were equivalent to water in fresh samples. However, only sixty components were identified in all the four parts, which might be caused by the low HS temperature (80 °C).

In the process of HS extraction, one of the key factors in HS extraction is matrix media. Among them, ionic liquids (ILs) are ideal matrix media due to their inherent properties, like negligible vapor pressure, high thermal stability, and excellent solubility [14,15]. ILs are composed of organic cations and inorganic anions, and they have received much attention in various applications, including synthesis, catalysis, and analytical chemistry [16,17,18]. When compared with traditional solvents, ILs have overcome the limitation of low HS temperature. Because of high boiling points of ILs, the sensitivity of detection can be improved by increasing the HS temperature. Thus, trace components with high boiling points can also be enriched and detected. Using ILs as matrix media, the volatile components in food and herbal medicines can be comprehensively and scientifically evaluated.

This study aims to establish an IL-based HS-GC-MS method to analyze and identify volatile components in leaf, pericarp, and seed of *Clausena lansium* from different areas in Hainan Province, China. As the key factor, different matrix media of HS were firstly optimized. An orthogonal design was then employed to assist in quickly and reliably finding other optimum extraction conditions. Cluster analysis was used to compare the correlation and difference between different parts and different areas. Because of the high boiling points of ILs, low-content components were better enriched, giving a comprehensive and accurate evaluation for the quality of *Clausena lansium*. This method is simple, rapid, accurate, sensitive, and suitable for the analysis and identification of volatile constituents in plant, food, and herbal medicines.

## 2. Material and Methods

### 2.1. Chemicals and Samples

Three ILs were obtained from Shanghai Cheng Jie Chemical Co. Ltd. (Shanghai, China), including 1-butyl-3-methylimidazolium hexafluorophosphate ([Bmim][PF_6_]), 1-butyl-3-methylimidazolium tetrafluoroborate ([Bmim][BF_4_]), and 1-butyl-3-methylimidazolium bis(trifluoromethane sulfonimide) ([Bmim][NTf_2_]). Prior to use, they were stirred at 110 °C overnight under vacuum to remove volatile impurities. C8–C40 n-alkanes were purchased from America o2si, number: CDGG-115320-05-1, 500 mg/L in hexane. The leaves and fruits of *Clausena lansium* were collected from Hainan Province, China in June 2017. The collection locations were Longquan town, Longhua District, Haikou City (HK1), Longqiao town, Longhua District, Haikou City (HK2), Longhua District, Haikou City (HK3), Xinglong town, Wanning City (WN1), Xinglong town, Wanning City (WN2), Yacheng District, Sanya City (SY1), Yacheng District, Sanya City (SY2), Qiongzhong Li & Miao Autonomous County (QZ), Qionghai City (QH), and Danzhou City (DZ). The leaves were with 5–11 leaflets; fruits were globose or ellipsoid; and, berries were approximately of 1.8–2.0 cm. All of the samples were identified by Professor Jianping Tian and were deposited in Public Research Laboratory, Hainan Medical University (Hainan, China). The leaf, pericarp, and seed of *Clausena lansium* employed in this study were abbreviated as L, P, and S, respectively. For example, leaf collected in HK1 was abbreviated as LHK1.

### 2.2. Instrumentation and Methods

#### 2.2.1. Instrumentation

Measurements were carried out on a Shimadzu GC-MS-QP2010-Plus system with electron impact ionization (EI). Data were processed and elaborated with Shimadzu GC-MS Solution software (Shimadzu, v 2.70, Kyoto, Japan), equipped with a Gerstel MPS2 Multi-Purpose Sampler (Tegent, Germany). The column was ZB-5MS fused silica (30 m × 2.5 mm; 0.25 μm film thickness) from Phenomenex, USA. Other instrumentation included FW-80 high speed grinder (Tianjin Taisite Instrument Co., LTD, Tianjin, China), AL104 electronic balance (METTLER TOLEDO Instrument Co., LTD, Shanghai, China), and 20 mL headspace vial equipped with PTFE/silicone rubber pad and magnetic cap (Agilent, California, CA, USA).

#### 2.2.2. GC-MS Analysis

Samples were analyzed by the GC-MS EI method with column ZB-5MS. Helium was used as a carrier gas at a flow rate of 1.0 mL/min in a split mode (1:20). The column temperature was maintained at 60 °C for 5 min and then programmed to 120 °C at a heating rate of 10 °C/min, further increased to 170 °C at a rate of 2 °C/min, and finally increased to 210 °C at a rate of 10 °C/min for remaining 10 min. Temperatures of both injector and connector were maintained at 270 °C. Operating parameters of MS: EI mode at 70 eV with a mass scanning range of 50–500 amu and source temperature of 250 °C. C8–C40 n-alkanes were used as reference points in the calculation of relative retention indexes (RIs). The percentage of compositions was obtained from the electronic integration of peak areas. Each compound was identified by comparing its retention index with n-alkanes and the NIST library database. Percentage of composition was computed from peak areas without applying correction factors.

#### 2.2.3. HS Extraction

The collected leaves of *Clausena lansium* were dried in shade. After removing pulps of fruits, pericarps and seeds were washed separately and then dried in an oven at 40–50 °C. The leaves, pericarps, and seeds were milled into powder with a grinder. 100 mg of sample powder and 2 mL of IL were added into a 20 mL headspace vial. The vial was closed with a PTFE/silicone rubber pad and magnetic cap, and it was equilibrated at 150 °C for 10 min. Finally, a 500 μL of injection volume from head space was injected into GC-MS for analysis.

## 3. Results and Discussion

### 3.1. Selection of Matrix Media

Rational selection of HS matrix media is the key in HS-GC-MS measurements. The HS efficiency of different matrix media was firstly investigated. For ILs, such as [Bmim][BF_4_], the equilibrium temperature can be reached to 300 °C, because of negligible vapor pressure and excellent thermal stability [19]. When considering that a high temperature may lead to a high pressure in the vial and the PTFE/silicone rubber pad must be used below the maximum operation temperature, 140 °C was chosen as the equilibrium temperature for three ILs in the pre-experiment. Whereas, the equilibrium temperature of water was set at 80 °C, since its boiling point is 100 °C. The equilibrium time was set at 30 min. Before sample analysis, blank matrix media were compared by using HS-GC-MS. The sample LHK1 was selected as an optimization object.

**Figure 1:**
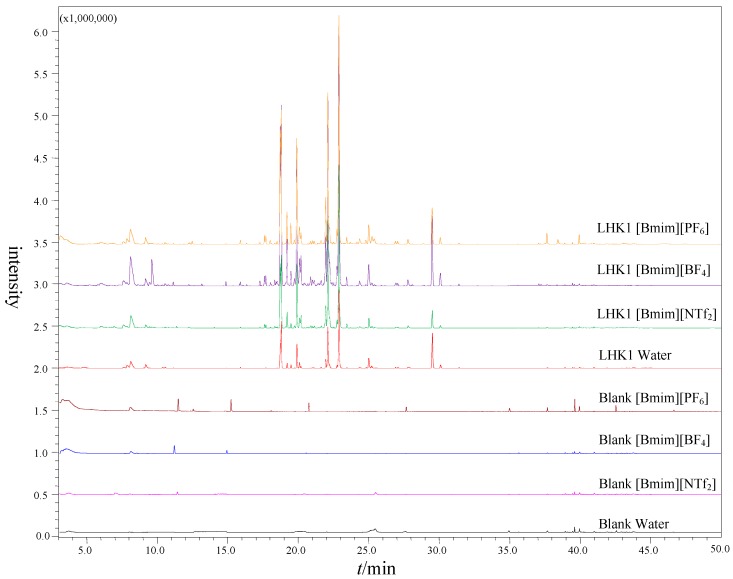
Chromatograms of blank matrix media and sample LHK1 when using three ILs and water as matrix media by headspace gas chromatography-mass spectrometry (HS-GC-MS).

Figure 1 shows GC-MS chromatograms of matrix media (three ILs and water) with and without sample LHK1. As shown in Figure 1, there are impurities in these four matrix media. The impurities may be organic solvents with low boiling point used in the synthesis of ILs. To remove impurities, ILs were stirred at 110 °C overnight under vacuum prior to use. Nevertheless, at high temperatures, even a trace amount of water can cause the release of hydrofluoric acid, which can corrode the glass and also increase the amounts of impurities. Besides, the rubber pad at high temperature may produce plasticizers. The peak area/number of impurities of these four matrix media is as follows: [Bmim][PF_6_] > [Bmim][BF_4_] > [Bmim][NTf_2_] > water. Supplemental Table S1 illustrates the comparison of peak area percentages and peak numbers in LHK1 using ILs and water as matrix media by HS-GC-MS. The results of impurities are consistent with the blank matrix media. When using [Bmim][PF_6_] as the matrix medium, the impurities of peak area percentage were up to 11.86%. There were less impurities when using [Bmim][NTf_2_], [Bmim][BF_4_], and water as matrix media. However, with a limited equilibrium temperature, when water was used as the matrix medium, the peak number detected was also the least, only 22. The peak number without impurities was 46 and 30 when using [Bmim][BF_4_] and [Bmim][NTf_2_], respectively. Therefore, [Bmim][BF_4_] was chosen as the matrix medium in the following study. The volatile components of pericarp and seed were also investigated while using these three ILs and water as matrix media by HS-GC-MS. The results were consistent with the sample LHK1.

### 3.2. Orthogonal Design for HS Optimization

After optimizing the HS efficiency of different matrix media, an orthogonal experimental design was used to investigate effects of three parameters on HS performance, including equilibrium temperature, equilibrium time, and stirring rate. Orthogonal experimental design is an efficient method for quickly generating useful information on key extraction variables. An OA_9_(3^3^) was built for the optimization of the main factors affecting the extraction efficiency. The factors and levels were equilibrium temperature (90 °C, 120 °C, 150 °C), equilibrium time (10 min, 20 min, 30 min), and stirring rate (0, 375 rpm, 750 rpm). According to the orthogonal design, nine experimental trials were conducted and randomly carried out. The total peak areas for volatile components without impurities were considered as the experimental response. The results of the sums of squares (*SS*) for different variables were calculated and are listed in Table 1. The aim was to determine which variable had predominant influence and to select the optimum extraction condition. The orthogonal design was analyzed by SPSS 17.0 (IBM Corp., Chicago, IL, USA).

It can be seen from the analysis of variance results in Table 1 that the order of the influence of each factor on extraction is equilibrium temperature > equilibrium time > stirring rate. Equilibrium temperature has the greatest influence on the extraction efficiency and it has the most statistically significant effect (*p* < 0.05). Two other factors have no significant effect on extraction efficiency. 

#### 3.2.1. Effect of Equilibrium Temperature

The equilibrium temperature has the greatest influence on the extraction efficiency. By increasing the temperature from 90 °C to 150 °C, the total peak area correspondingly increased greatly. For equilibration temperatures higher than 150 °C, they resulted in too many impurities. For this reason, the final equilibrium temperature was set at 150 °C.

#### 3.2.2. Effect of Equilibrium Time

To optimize the equilibrium time, the sample solution was heated at different times, ranging from 10 to 30 min. The efficiency of extraction slightly declined when the equilibrium time was increased from 10 to 30 min. The results indicate that 10 min was enough for reaching the equilibration of extraction. This may be due to the small amount of matrix medium (2 mL) in HS, and an equilibration can be achieved in a short time. Based on this result, 10 min was selected for subsequent studies.

#### 3.2.3. Effect of Stirring Rate

Usually, mass transfer from a liquid sample to the HS can be increased by stirring the sample. However, in this study, there was no significant change in the extraction efficiency by varying the stirring rate from 0 to 750 rpm. This result might be caused by rapid reaching of equilibration because of high equilibrium temperature and small amount of matrix medium. Since the stirring rate had no notable effects on extraction efficiency, it was not considered to be a key factor for further studies.

### 3.3. Analysis of Samples

**Figure 2:**
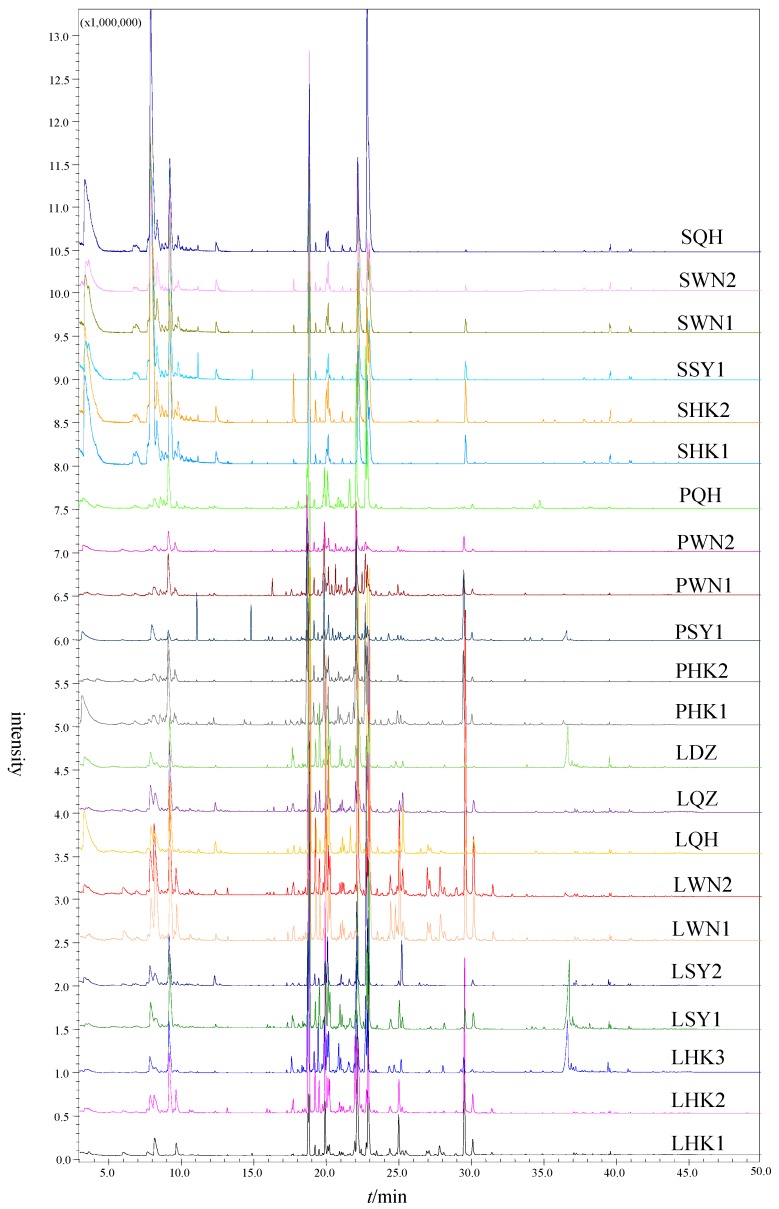
Chromatograms of leaves, pericarps, and seed of *Clausena lansium* from different areas by ionic liquids-headspace gas chromatography-mass spectrometry (IL-HS-GC-MS).

The volatile components of samples were analyzed after optimization. The GC-MS chromatograms of leaves, pericarps, and seeds are shown in Figure 2. Components were identified by comparing their RIs with standard compounds, and matching their fragmentation patterns of mass spectra with published mass spectra. All of the volatile chemical components in the *Clausena lansium* are summarized in Table 2.

The number of components was different in different parts of *Clausena lansium.* Ninety components in total were detected, among them, seventy-six in leaves, seventy-nine in pericarps, and thirty-three in seeds. Based on the percentages of volatile chemical components in various parts, twenty-two samples were analyzed by cluster analysis (SPSS 17.0).

**Figure 3:**
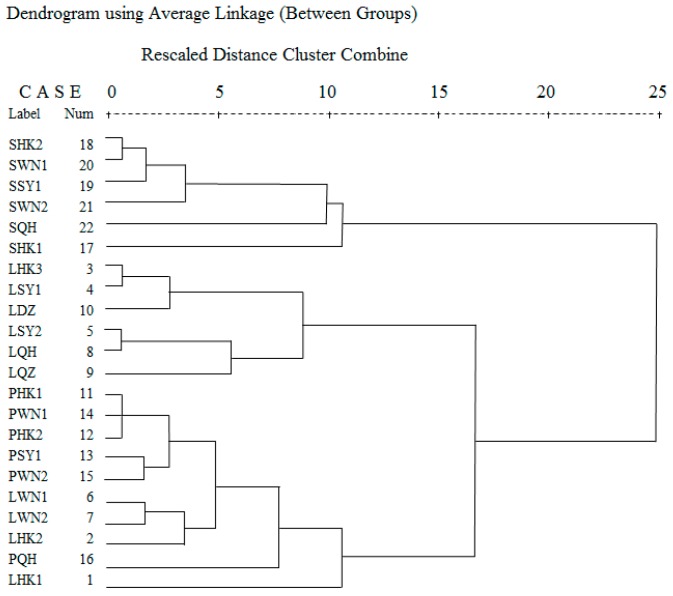
Dendrograms based on chemical compositions of seeds of various parts of *Clausena lansium* from different areas.

The clustering results are shown in the Figure 3. The samples were clustered into two groups, the seeds and the others. This result indicates that volatile chemical components in seeds were different from that in leaves and pericarps. There were many common components and similar relative percentages in the leaves and pericarps. Based on the cluster analysis, six clusters were classified. Table 3 shows the variation of some important volatile components (%) of *Clausena lansium* in different clusters.

Cluster I–VI are listed as below:

Cluster I: sabinene & β-bisabolene. SHK2, SWN1, SSY1, SWN2, and SQH belonged to this cluster. All these samples were seeds. The volatile chemical components were rich in sabinene (17.76–27.53%), and accompanied by β-bisabolene (8.16–22.25%) and caryophyllene (9.59–16.62%).

Cluster II: sabinene & β-sesquiphellandrene. Only one sample was in this cluster, SHK1. The percentage of sabinene was high at 43.81%, and β-sesquiphellandrene was 22.25%. The seeds from HK1 were significantly different from that of other areas.

Cluster III: caryophyllene. LHK3, LSY1, LDZ LSY2, LQH, and LQZ were classified into this cluster. They contained more caryophyllene, a relative percentage of 17.64–33.28%. Percentages of β-sesquiphellandrene, β-bisabolene, and α-bergamotene were also relatively large.

Cluster IV: β-bisabolene & *cis*-α-santalol. There were PHK1, PHK2, PSY1, PWN1, PWN2, LWN1, LWN2, and LHK2 in this cluster. No component in this cluster had a percentage higher than 20%. The percentages of components in the three samples are similar, with a value of approximately 10%. They mainly include β-bisabolene, β-sesquiphellandrene, *cis*-α-santalol, caryophyllene, (E)-β-farnesene, and elixene. 

Cluster V: β-bisabolene & β-sesquiphellandrene. Only PQH was in this cluster. The percentages of β-bisabolene and β-sesquiphellandrene were 15.33% and 15.28%, respectively.

Cluster VI: α-bergamotene & β-bisabolene. There was only LHK1 in this cluster. The main components were α-bergamotene (21.39%) and β-bisabolene (17.52%). It was different from Cluster V (PQH), because the percentage of β-sesquiphellandrene in LHK1 was only 1.21%. In addition, the percentage of *cis*-α-santalol in LHK1 was 10.65%, however, it was only 0.18% in PQH.

Through the cluster analysis, we found that the volatile components of different parts were different, with a greater difference in seeds than in leaves and pericarps. The contents of monoterpenes in seed were higher than that in leaves and pericarps. The main compositions of leaves and pericarps were sesquiterpenes. Leaves, pericarps, and seeds of HK2, SY1, WN1, and WN2 were all divided into the same clusters, respectively. This result indicates that the components of these areas had a lot in common. Leaf and seed of HK1 were different from that of all other areas, but the pericarp of HK1 was similar to that of WN1, HK2, SY1, and WN2. There are many cultivars of *Clausena lansium*. In terms of fruit shapes, they are roughly round, elliptical, and broad-oval. We reason that besides different areas, growth conditions, ages, and other factors, different cultivars may also be one of the important factors affecting the composition and percentage of volatile components.

### 3.4. Method Performance Comparison

In Table 4, the present method was compared with some of the reported methods for the extraction and analysis of volatile components of *Clausena lansium*. In three previous studies, the essential oil was analyzed by hydrodistillation, followed by GC-MS in Hainan, China [7], Thailand [8], and Cuba [9]. The extraction time was 3 h, 6 h, and 4 h in these three studies, respectively. Thirty-two components in leaf and twenty-four components in seed were identified for samples from Hainan. The main components identified in the leaf oil were β-santalol (35.2%) and bisabolol (13.7%), while in the seed oils were phellandrene (54.8%) and limonene (23.6%). In both fresh and dried fruit samples from Thailand, fifty-three components were identified, and the main components were sabinene (33.68–66.73%), α-pinene (9.57–13.35%), and 1-phellandrene (5.77–10.76%). For leaf samples from Cuba, seventy compounds were identified. The most prominent components were caryophyllene oxide (16.8%) and (*Z*)-α-santalol (11.7%). The components and percentages were different from different areas, and the differences were much greater in different parts of *Clausena lansium*. SFE was used in the extraction of *Clausena lansium* leaf from Guangdong, China [10]. Thirty-six components were identified by GC-MS after 4 h of extraction of 200 g samples. The main components were 4-terpineol (26.94%) and γ-terpinene (14.39%). The components and percentages that were obtained by SFE were also different from that by hydrodistillation. These traditional extractions had notable shortcomings, including requiring large amount of sample, tedious process, and time-consuming operation.

Chokeprasert et al. [13] analyzed the volatile components of 1 g fresh leaf, skin, and seed in Thailand by HS-GC-MS. Thirty-nine components were identified in the leaf, and the major components were sabinene (15%) and β-bisabolene (9.88%); thirty components were identified in the skin, and the major components were sabinene (69.07%) and α-phellandrene (10.63%); and, twenty-five components were identified in the seed, and the major components was sabinene (83.56%). For those high-percentage components, they were roughly equivalent to our results. However, their percentages were in greater difference than our results. It maybe because components with low boiling points volatilized easier in the HS with a temperature of 80 °C, but some components with high-boiling points, such as spathulenol, caryophyllene oxide, even phytone, were not volatilized to the headspace and thus were not detected. ILs, as a group of novel solvents, are used as ideal matrix media due to their inherent properties, including negligible vapor pressure, high thermal stability, and excellent solubility. In our group, we have applied ILs as headspace matrix media for analyzing residuals in pharmaceuticals [20,21]. In our current study, more volatile components, including components with high-boiling points and low content, were detected using only 100 mg of sample by [Bmim][BF_4_] based HS-GC-MS. Using ILs as HS matrix media with high boiling points, the sensitivity can be improved by increasing the HS equilibrium temperature to enrich the trace components with high boiling points. Moreover, due to the high temperature of equilibrium and small amount of matrix medium, the equilibration was easily achieved in only 10 min. The current method is simple, rapid, accurate, and sensitive. It is suitable for the analysis and identification of volatile components of different parts of *Clausena lansium* from different areas in Hainan.

## 4. Conclusions

In this study, a simple, rapid, sensitive, and precise IL-HS-GC-MS method was developed to identify and analyze the volatile components of different parts of *Clausena lansium* from different areas in Hainan for the first time. Ninety components, including components with trace amounts and high-boiling points, were extracted in a short time. The method is highly efficient and sensitive. It may provide a new way of scientific analysis and evaluation for the quality control of plant, food, and herbal medicines.

## Figures and Tables

**Table 1:** Analysis of variance results for experimental response in the OA_9_(3^3^) matrix.

Source	III Square Sum	*df*	*SS*	*F*	*Sig.*
Equilibrium temperature	4.034 × 10^15^	2	2.017 × 10^15^	780.793	0.001
Equilibrium time	3.934 × 10^13^	2	1.967 × 10^13^	7.614	0.116
Stirring rate	4.051 × 10^12^	2	2.025 × 10^12^	0.784	0.561
Error	5.166 × 10^12^	2	2.583 × 10^12^		
Total	1.280 × 10^16^	9			

*df =* degrees of freedom; *SS =* Sum of squares; *F =* critical value is 19.00 (*p* < 0.05).

**Table 2:** Volatile chemical components identified in the *Clausena lansium* from different areas by IL-HS-GC-MS.

No.	Components	Molecular Formula	Retention Indexe ^a^	Relative Percentage/%																					
				LHK1	LHK2	LHK3	LSY1	LSY2	LWN1	LWN2	LQH	LQZ	LDZ	PHK1	PHK2	PSY1	PWN1	PWN2	PQH	SHK1	SHK2	SSY1	SWN1	SWN2	SQH
1	2-hexenal	C_6_H_10_O		-	-	-	-	-	-	-	-	-	-	-	1.35	-	0.56	-	0.97	-	-	-	-	-	-
2	α-thujene	C_10_H_16_		-	-	-	-	0.13	-	-	-	-	-	-	-	-	-	-	0.19	0.54	0.4	0.6	0.57	0.42	0.38
3	pinene	C_10_H_16_		-	-	-	-	0.16	0.15	-	-	-	-	0.71	1.17	-	0.76	-	0.87	0.89	0.75	0.89	0.87	0.53	0.55
4	benzaldehyde	C_7_H_6_O		1.04	0.48	-	-	0.27	0.3	-	0.19	-	-	0.2	0.39	-	-	0.49	-	0.98	0.78	1.53	-	0.51	1.08
5	sabinene	C_10_H_16_		0.13	2.42	2.51	2.33	3.14	2.24	2.68	1.47	3.45	1.83	0.71	0.71	0.25	0.37	0.51	0.59	43.81	25.38	27.49	23.57	17.76	27.53
6	6-methyl-5-hepten-2-one	C_8_H_14_O		4.38	2.94	0.4	0.72	2.75	5.73	5.01	1.17	3.31	0.42	0.91	1.78	-	2.76	1.97	-	-	-	-	-	-	-
7	myrcene	C_10_H_16_		-	-	0.62	-	-	-	-	-	-	-	1.26	1.87	-	-	2.77	3.05	2.89	3.47	3.72	3.53	4.08	3.24
8	β-phellandrene	C_10_H_16_		0.16	0.13	0.16	0.22	0.19	0.14	0.13	-	0.21	0.12	1.27	1.46	0.34	1.04	0.97	2.04	0.31	0.93	0.72	0.49	0.46	0.53
9	α-terpinen	C_10_H_16_		-	-	-	-	0.13	0.08	0.1	-	-	-	0.66	0.7	0.28	0.53	0.46	1.29	0.42	0.67	0.6	0.28	0.25	0.53
10	p-cymene	C_10_H_14_		-	0.13	0.11	0.1	0.19	0.11	0.11	0.09	0.11	0.07	0.59	0.52	0.16	0.44	0.34	0.52	-	-	-	-	-	-
11	α-phellandrene	C_10_H_16_		0.1	6.23	6.45	6.6	7.36	5.48	5.63	4.38	7.5	5.82	8.93	11.23	2.02	7.9	8.38	9.23	9.27	8.03	0.69	11.52	12.67	7.35
12	benzeneacetaldehyde	C_8_H_8_O		0.15	0.26	-	0.27	0.47	0.27	0.18	-	0.17	-	0.4	0.82	-	0.49	0.68	0.1	0.41	0.86	1.32	0.7	0.62	0.61
13	melonal	C_9_H_16_O	806	1.87	2.15	-	-	-	1.53	0.97	-	0.3	-	0.89	2.06	0.18	1.04	3.78	0.1	-	-	-	-	-	-
14	γ-terpinen	C_10_H_16_	808	-	-	0.13	0.11	0.39	-	-	0.11	0.32	-	0.66	1.17	0.28	0.66	-	0.9	0.92	1.19	-	1.13	1.17	1.03
15	terpinolen	C_10_H_16_	869	-	-	-	-	0.07	-	-	-	-	-	0.12	0.13	-	0.08	-	0.3	0.14	0.2	0.2	-	-	-
16	linalol	C_10_H_18_O	899	0.05	0.23	-	-	-	0.17	0.22	-	0.21	-	0.16	0.13	0.06	-	-	0.09	-	0.2	-	-	-	-
17	borneol	C_10_H_16_O	973	-	-	-	-	-	-	-	-	-	-	0.06	-	0.04	0.03	-	-	-	-	-	-	-	-
18	4-terpinenol	C_10_H_18_O	981	-	-	-	-	-	-	-	-	-	-	0.08	0.04	0.05	-	-	0.07	-	-	-	-	-	-
19	*cis*-piperitol	C_10_H_18_O	1009	-	-	-	-	0.2	-	-	0.1	0.16	-	-	-	-	-	-	-	-	-	-	-	-	-
20	(*E*)-citral	C_10_H_16_O	1065	-	-	-	-	-	-	-	-	-	-	-	-	0.07	-	-	-	-	-	-	-	-	-
21	phellandral	C_10_H_16_O	1078	-	-	-	-	-	-	-	-	-	-	0.31	0.05	0.23	0.06	-	0.02	-	-	-	-	-	-
22	bornyl acetate	C_12_H_20_O_2_	1083	-	-	-	-	-	-	-	-	-	-	0.11	0.03	0.04	0.04	-	0.06	-	-	-	-	-	-
23	α-santalene	C_15_H_24_	1128	0.03	0.22	0.03	0.1	-	0.02	0.05	0.04	0.05	0.12	0.04	-	0.09	-	-	-	-	0.04	-	-	0.07	-
24	δ-elemene	C_15_H_24_	1136	0.05	0.12	0.05	0.05	-	-	0.06	-	-	-	0.1	-	0.38	0.06	0.07	-	-	-	-	-	-	-
25	α-cubebene	C_15_H_24_	1145	0.09	0.03	0.03	0.05	0.05	0.05	0.06	0.04	0.2	-	0.16	0.12	0.33	1.25	0.34	0.04	-	-	-	-	-	-
26	copaene	C_15_H_24_	1177	0.14	0.1	0.16	0.2	0.17	0.16	0.16	0.13	0.23	0.13	0.23	0.14	0.26	0.31	0.2	0.14	-	-	-	-	-	-
27	bergamotene	C_15_H_24_	1186	0.1	0.22	1.22	0.59	-	-	0.15	-	-	1.08	0.28	0.23	0.48	0.74	0.38	0.06	-	-	-	-	-	-
28	β-elemen	C_15_H_24_	1189	0.13	0.7	-	0.32	0.28	0.61	0.43	0.24	0.89	0.69	0.22	0.29	-	-	-	0.03	0.07	1.28	0.03	0.23	0.52	-
29	(−)-β-santalene	C_15_H_24_O	1195	-	-	-	-	-	-	-	-	-	-	-	-	-	-	-	-	-	0.08	-	-	0.05	-
30	zingiberene	C_15_H_24_	1202	-	-	0.1	0.09	0.25	0.05	-	0.13	0.08	0.09	0.19	-	0.09	0.12	0.04	0.66	-	-	-	-	-	-
31	α-cedrene	C_15_H_24_	1208	0.08	0.14	0.37	0.2	-	0.09	0.08	-	0.05	0.17	0.35	0.33	0.34	0.32	0.25	0.13	-	-	-	-	-	-
32	α-chamigrene	C_15_H_24_	1211	0.07	0.08	0.29	0.16	-	0.06	-	-	0.04	0.11	0.21	0.22	0.21	0.24	0.14	0.03	-	-	-	-	-	-
33	(−)-*trans*-α-bergamotene	C_15_H_24_	1212	0.04	0.22	0.11	0.1	0.06	0.19	0.2	0.07	0.1	0.11	0.09	0.13	0.17	-	0.17	0.04	-	-	-	-	-	-
34	elixene	C_15_H_24_	1218	3.24	9.13	7.33	7.14	-	6.98	10.92	-	-	7.64	5.15	5.7	11.68	7.47	11.94	2.94	-	-	-	-	-	-
35	caryophyllene	C_15_H_24_	1220	4.96	13.7	17.64	21.68	31.06	11.4	9.71	31.69	33.28	30.14	4.65	8.96	1.62	4.34	3.02	5.67	6.75	12.6	9.59	11.1	16.62	7.16
36	β-cedrene	C_15_H_24_	1225	0.07	0.03	0.14	0.1	-	0.04	-	-	-	0.05	0.07	0.1	0.05	0.09	0.05	0.08	-	-	-	-	-	-
37	(+)-α-longipinene	C_15_H_24_	1230	0.87	2.21	1.38	1.31	0.86	1.96	2.02	0.89	1.31	1.46	1.17	1.97	2.27	1.98	2.15	0.87	0.21	0.59	0.3	0.37	0.42	0.31
38	alloaromadendrene	C_15_H_24_	1237	0.56	1.54	2.83	1.95	0.56	1.29	0.93	0.43	1.21	3.52	0.59	0.57	0.8	0.59	0.79	0.09	0.07	0.12	0.03	0.1	0.21	0.03
39	β-santalene	C_15_H_24_	1244	0.26	0.47	0.61	0.48	0.13	0.32	0.34	0.12	0.25	0.43	0.4	0.5	0.55	0.46	0.5	0.22	-	0.02	-	-	-	-
40	isoledene	C_15_H_24_	1247	-	-	-	-	-	-	-	-	-	-	-	-	-	1.32	1.01	1.03	-	-	-	-	-	-
41	(*E*)-β-farnesene	C_15_H_24_	1249	4.91	11.68	4.2	3.51	2.48	7.09	5.54	3.52	2.94	3.22	5.5	7.71	7.05	6.52	8	4.03	0.49	1.22	0.43	0.88	0.94	0.92
42	humulene	C_15_H_24_	1253	1.01	2.15	2.83	3.11	3.92	1.69	1.46	3.23	3.57	3.66	1.52	2.01	0.67	1.62	1.48	3.82	0.81	1.39	1.13	1.35	1.86	0.83
43	α-himachalene	C_15_H_24_	1256	1.07	1.98	2.64	1.75	-	1.23	1.05	0.31	0.71	1.84	2.33	3.43	2.59	2.96	3.11	0.94	0.14	0.43	0.17	0.21	0.32	0.16
44	bicyclosesquiphellandrene	C_15_H_24_	1261	0.17	0.11	0.1	0.1	-	0.06	-	-	-	0.04	0.11	-	0.07	0.21	0.06	0.09	-	-	-	-	-	-
45	ionene	C_13_H_18_	1263	-	-	-	-	-	-	-	-	-	-	-	0.73	1.45	1.17	0.51	0.12	-	-	-	-	-	-
46	(−)-β-cadinene	C_15_H_24_	1271	0.03	0.11	0.16	0.14	-	0.08	0.06	-	0.12	0.1	0.26	0.47	0.56	2.83	1.61	0.45	-	-	-	-	-	-
47	γ-muurolene	C_15_H_24_	1275	-	0.73	-	0.21	0.09	0.08	0.09	-	0.21	0.12	-	-	-	0.56	0.32	-	-	-	-	-	-	-
48	β-himachalene	C_15_H_24_	1278	0.44	0.41	2.23	1.18	0.17	0.41	0.32	0.14	0.45	1.19	1.45	1.65	1.23	1.16	0.66	1.51	-	-	-	-	-	-
49	curcumene	C_15_H_24_	1280	0.36	0.4	1.09	0.77	0.91	0.53	0.41	0.52	0.75	0.64	0.8	1.49	0.91	-	1.75	0.81	0.12	0.26	0.12	0.36	0.24	0.22
50	(*Z*)-β-farnesene	C_15_H_24_	1281	0.32	0.4	0.39	0.27	0.28	0.35	0.36	0.26	0.23	0.28	0.58	-	0.57	1.99	-	0.5	-	0.03	-	-	-	0.07
51	cubebene	C_15_H_24_	1287	0.12	0.19	0.09	0.17	0.08	0.08	-	0.07	0.1	0.1	0.2	0.52	0.28	0.37	0.15	-	-	-	-	-	-	-
52	β-selinene	C_15_H_24_	1293	-	0.15	-	0.23	-	0.14	0.09	-	0.16	-	0.22	0.39	0.43	1.92	1.07	0.09	-	-	-	-	-	-
53	γ-elemene	C_15_H_24_	1294	0.32	0.6	1.07	1.02	0.75	0.35	0.14	1.06	0.39	1.11	0.55	-	-	-	-	1.17	-	-	-	-	-	-
54	epizonarene	C_15_H_24_	1296	-	-	0.53	-	-	-	0.1	-	-	-	0.81	1.32	0.88	0.93	0.59	2.57	0.04	0.12	-	0.06	0.13	0.17
55	α-muurolene	C_15_H_24_	1297	-	-	-	-	-	-	-	-	-	-	-	-	-	0.4	0.11	0.12	-	-	-	-	-	-
56	β-vatirenene	C_15_H_22_	1301	-	-	-	-	-	-	-	-	-	-	-	-	-	0.53	0.24	-	-	-	-	-	-	-
57	α-farnesene	C_15_H_24_	1303	1.88	4.7	0.66	0.98	0.38	0.28	0.15	0.35	1.96	1.71	1.58	2.78	0.72	1.11	-	0.45	0.03	-	-	-	-	-
58	β-bisabolene	C_15_H_24_	1306	17.52	4.16	12.12	9.73	7.02	13.81	10.5	8.94	11.46	9.77	12.56	8.56	12.11	12.59	13.7	15.33	5.83	8.16	9.33	11.85	15.19	5.65
59	(+)-α-longipinene	C_15_H_24_	1309	-	4.89	-	-	-	-	-	-	-	2.93	-	-	-	-	-	-	-	-	-	-	-	-
60	δ-cadinene	C_15_H_24_	1315	0.17	0.22	0.25	0.35	0.13	0.19	0.15	0.15	0.45	0.19	0.4	0.62	0.76	2.42	3.28	0.15	0.02	0.04	-	-	0.05	-
61	β-sesquiphellandrene	C_15_H_24_	1324	1.21	1.52	5.93	4.34	19.8	2.51	1.43	18.93	4.15	2.53	8.11	5.56	2.51	5.81	2.73	15.28	3.2	4.28	3.35	4.7	4.08	22.25
62	α-bergamotene	C_15_H_24_	1325	21.39	4.28	4.57	8.41	5.8	10.64	7.9	9.91	11.1	3.47	5.66	5.38	2.25	3.54	-	8.64	2.63	4.87	4.79	6.98	5.35	-
63	cubinene	C_15_H_24_	1330	-	-	-	-	-	-	-	-	-	-	-	-	0.21	0.72	-	-	-	-	-	-	-	-
64	germacrene D	C_15_H_24_	1335	-	-	0.08	0.07	-	0.06	-	-	-	0.04	-	-	-	-	-	-	-	-	-	-	-	-
65	α-bisabolene	C_15_H_24_	1336	0.44	0.11	0.2	0.19	0.13	0.24	0.18	0.21	0.19	0.15	0.35	0.22	0.45	0.61	0.31	0.44	-	-	-	-	-	-
66	*trans*-nerolidol	C_15_H_26_O	1357	1.19	0.62	0.87	0.87	-	1.52	0.81	0.07	0.38	0.25	0.64	-	1.2	0.84	-	-	-	-	-	-	-	-
67	dendrolasin	C_15_H_22_O	1366	0.34	-	0.9	0.12	-	1	0.12	0.11	0.22	0.6	0.16	-	0.28	0.42	-	-	-	-	-	-	-	-
68	spathulenol	C_15_H_24_O	1372	4.5	2.34	0.15	1.74	0.42	2.9	2.96	0.47	0.95	-	1.06	1.19	0.75	1.59	1.54	-	-	-	-	-	-	-
69	caryophyllene oxide	C_15_H_24_O	1377	0.48	0.4	1.12	0.77	3.82	0.84	1.05	1.8	1.39	0.53	0.58	0.25	0.64	0.42	0.38	0.11	-	-	-	-	-	-
70	humuleneepoxide II	C_15_H_24_O	1405	-	-	0.03	-	0.23	0.04	0.07	0.15	0.05	-	-	-	-	-	-	-	-	-	-	-	-	-
71	*cis*-Z-α-bisabolene epoxide	C_15_H_24_O	1416	0.48	-	-	0.09	0.11	0.67	1.12	0.42	0.1	-	-	-	-	-	-	-	-	-	-	-	-	-
72	cedrol	C_15_H_26_O	1420	0.87	0.05	0.21	0.17	0.03	0.6	0.69	0.26	0.08	-	0.23	-	0.24	0.21	-	0.15	-	-	-	-	-	-
73	cubenol	C_15_H_24_O	1428	0.13	0.07	-	-	-	0.1	0.12	-	-	-	-	-	-	-	-	-	-	-	-	-	-	-
74	(*E*)-nuciferol	C_15_H_20_	1435	1.69	-	-	-	0.05	1.27	1.34	0.15	0.08	-	-	-	-	-	-	-	-	-	-	-	-	-
75	(+)-γ-curjunene	C_15_H_24_	1442	0.46	0.13	0.63	0.41	-	0.35	0.39	-	0.06	0.2	0.34	0.07	0.41	0.23	-	-	-	-	-	-	-	-
76	α-cadinol	C_15_H_24_O	1450	-	-	-	-	-	-	-	-	-	-	-	-	0.06	0.07	-	-	-	-	-	-	-	-
77	*cis*-α-santalol	C_15_H_24_O	1477	10.65	10.35	1.24	-	-	5.98	13.93	0.51	0.93	0.53	5.32	5.41	9.36	3.08	4.66	0.18	1.07	1.76	1.04	0.71	0.34	0.08
78	α-bisabolol	C_15_H_26_O	1486	2.34	1.4	0.29	1.24	0.69	1.87	2.73	0.73	1.2	0.14	0.96	1.08	1.63	0.96	0.93	0.6	-	-	-	-	-	-
79	α-santalol	C_15_H_24_O	1513	0.3	0.32	-	-	-	0.37	0.43	-	-	-	0.16	0.25	0.2	0.13	0.1	-	-	-	-	-	-	-
80	(*Z*,*E*)-a-sarnesene	C_15_H_24_	1541	0.04	-	-	-	-	0.05	0.09	-	-	-	-	-	-	-	-	-	-	-	-	-	-	-
81	ledol	C_15_H_26_O	1562	0.06	-	0.06	0.04	-	0.08	0.08	-	-	0.19	0.17	0.3	0.37	0.21	0.1	-	-	-	-	-	-	-
82	*cis*-a-bisabolene	C_15_H_24_	1632	-	-	7.98	7.84	-	-	-	-	-	5.21	-	-	2.43	-	-	-	-	-	-	-	-	-
83	santalol	C_15_H_24_O	1641	-	-	0.12	0.14	0.11	-	0.24	-	0.14	-	0.37	0.08	-	0.16	-	-	-	-	-	-	-	-
84	*trans*-Z-a-bisabolene epoxide	C_15_H_24_O	1644	-	-	0.72	0.78	-	-	-	-	-	0.62	-	-	0.27	-	-	-	-	-	-	-	-	-
85	pentadecanal	C_15_H_30_O	1645	0.19	-	0.35	0.35	0.2	0.08	0.08	0.07	0.18	0.27	-	-	-	-	-	-	-	-	-	-	-	-
86	phytone	C_18_H_36_O	1650	0.17	-	0.34	0.31	0.41	0.08	0.07	0.05	0.14	0.21	-	-	-	-	-	-	-	-	-	-	-	-
87	lanceol	C_15_H_24_O	1661	-	-	-	0.3	-	-	-	0.04	0.05	-	-	-	-	-	-	0.1	-	-	-	-	-	-
88	farnesyl acetone	C_18_H_30_O	1706	-	-	-	-	-	-	0.03	-	-	-	-	-	-	-	-	-	-	-	-	-	-	-
89	methyl palmitate	C_17_H_34_O_2_	1725	0.08	0.06	0.47	0.25	0.36	0.09	0.08	0.09	0.18	0.53	0.05	0.06	-	0.08	-	0.05	0.14	0.12	0.23	0.36	0.16	0.11
90	ethyl palmitate	C_18_H_36_O_2_	1791	0.04	-	0.19	0.12	0.1	0.04	-	0.05	0.11	0.13	-	0.09	-	-	-	0.02	0.06	0.03	0.13	0.25	-	0.07
Total				93.94	98.41	97.48	96.94	97.34	97.17	96.5	93.86	98.61	96.5	84.96	95.14	76.86	93.86	88.79	89.17	82.26	80.3	68.43	82.17	85.02	80.86

^a^ Retention Indexe: calculated Retention Indexe.

**Table 3:** Variation of some important volatile components (%) of *Clausena lansium* in different clusters.

Components	Cluster I					Cluster II	Cluster III							Cluster IV							Cluster V	Cluster VI
	SHK2	SWN1	SSY1	SWN2	SQH	SHK1	LHK3	LSY1	LDZ	LSY2	LQH	LQZ	PHK1	PWN1	PHK2	PSY1	PWN2	LWN1	LWN2	LHK2	PQH	LHK1
sabinene	**25.38**	**23.57**	**27.49**	**17.76**	**27.53**	**43.81**	2.51	2.33	1.83	3.14	1.47	3.45	0.71	0.37	0.71	0.25	0.51	2.24	2.68	2.42	0.59	0.13
β-bisabolene	**8.16**	**11.85**	**9.33**	**15.19**	**22.25**	5.83	12.12	9.73	9.77	7.02	8.94	11.46	**12.56**	**12.59**	**8.56**	**12.11**	**13.7**	**13.81**	**10.5**	**4.16**	**15.33**	**17.52**
caryophyllene	12.6	11.1	9.59	16.62	7.16	6.75	**17.64**	**21.68**	**30.14**	**31.06**	**31.69**	**33.28**	4.65	4.34	8.96	1.62	3.02	11.4	9.71	13.7	5.67	4.96
elixene	-	-	-	-	-	-	7.33	7.14	7.64	-	-	-	5.15	7.47	5.7	11.68	11.94	6.98	10.92	9.13	2.94	3.24
β-sesquiphellandrene	3.2	3.35	4.28	4.7	4.08	**22.25**	5.93	19.8	2.53	19.8	18.93	4.15	8.11	5.81	5.56	2.51	2.73	4.34	19.83	1.52	**15.28**	1.21
α-bergamotene	2.63	4.79	4.87	6.98	5.35	-	4.57	5.8	3.47	5.8	9.91	11.1	5.66	3.54	5.38	2.25	-	8.41	5.8	4.28	8.64	**21.39**
*cis*-α-santalol	1.07	1.04	1.76	0.71	0.34	0.08	1.24	-	0.51	-	0.93	0.53	**5.32**	**3.08**	**5.41**	**9.36**	**4.66**	**5.98**	**13.93**	**10.35**	0.18	10.65

Bold values indicate the values of the dominated components in each cluster. -: Not detected.

**Table 4:** Comparisons for volatile components of *Clausena lansium* between the current method and other previously reported methods.

Pretreatment Method	Analysis Method	Source	Part	Pretreatment Time	Sample Amount	Identified Components Number	Ref.
Hydrodistillation	GC-MS	Hainan, China	Leaf, seed	3 h	-	32, 24	[7]
Hydrodistillation	GC-MS	Thailand	Fruit	6 h	500 g (fresh) or 200 g (dried)	53	[8]
Hydrodistillation	GC-FID ^a^, GC-MS	Cuba	Leaf	4 h	-	70	[9]
SFE	GC-MS	Guangdong, China	Leaf	4 h	200 g	36	[10]
Headspace (water as matrix medium)	GC-MS	Thailand	Leaf, skin, seed	20 min	1 g (fresh)	39, 30, 25	[13]
Headspace (ionic liquid as matrix medium)	GC-MS	Hainan, China	Leaf, pericarp, seed	10 min	100 mg	76, 79, 33	This work

-: not mentioned. ^a^ FID: Flame Ionization Detector.

## Supplementary Materials

The supplementary materials are available online.

## Abbreviations

IL	ionic liquid
HS	headspace
GC-MS	gas chromatography-mass spectrometry
SFE	supercritical fluid extraction
[Bmim][PF_6_]	1-butyl-3-methylimidazolium hexafluorophosphate
[Bmim][BF_4_]	1-butyl-3-methylimidazolium tetrafluoroborate
[Bmim][NTf_2_]	1-butyl-3-methylimidazolium bis(trifluoromethane sulfonimide)
EI	electron impact ionization
RIs	retention indexes
*df*	degrees of freedom
*SS*	sum of squares
OA	orthogonal arrays
FID	flame ionization detector
