# Supplementary material for: Rapid and Sensitive Analysis of Volatile Components of Different Parts of Clausena lansium by Ionic Liquid Based Headspace Gas Chromatography-Mass Spectrometry
Source: Molecules. 2018 Dec 27;24(1):91. doi: 10.3390/molecules24010091 (PMC6337593; doi:10.3390/molecules24010091)
Supplement: Supplementary file 1 [file molecules-24-00091-s001.pdf]

Rapid and Sensitive Analysis of Volatile Components of Different Parts of *Clausena lansium* by Ionic Liquid Based Headspace Gas Chromatography-Mass Spectrometry

Xiaowen He [1,2,†], Yinzheng Ma [2,†], Guohui Yi [3], Yingying Wen [4], Yunxia Zhang [3], Haiying Zhang [3], Lantong Zhang [1,*]

Supplementary Material

Table S1. Comparison of peak area percentages and peak numbers in LHK1 when using ILs and water as matrix media by HS-GC-MS.

Index	Types of matrix media			
	**[Bmim][PF_6]**	**[Bmim][BF_4]**	**[Bmim][NTf_2]**	**Water**
Total peak area percentage/ Total peak number	100.00%/59	100.00%/84	100.00%/54	100.00%/37
Identified peak area percentage/ Identified peak number	98.07%/52	96.77%/62	97.44%/41	97.05%/31
Peak area of impurities/ Peak number of impurities	11.86%/17	3.73%/16	3.14%/11	2.95%/9
Peak area without impurities/ Peak number without impurities	86.21%/35	92.93%/46	94.30%/30	94.10%/22
